# Health related quality of life of Dutch children: psychometric properties of the PedsQL in the Netherlands

**DOI:** 10.1186/1471-2431-9-68

**Published:** 2009-11-03

**Authors:** Vivian Engelen, Marleen M Haentjens, Symone B Detmar, Hendrik M Koopman, Martha A Grootenhuis

**Affiliations:** 1 Psychosocial Department, Academic Medical Centre/Emma Children's Hospital, Amsterdam, The Netherlands; 2 Department of Education, University of Amsterdam, Amsterdam, The Netherlands; 3 Prevention and Health Department, TNO, Leiden, The Netherlands; 4 Medical Psychology Department, Leiden University Medical Center, Leiden, The Netherlands

## Abstract

**Background:**

Knowledge about psychometric properties of the Pediatric Quality of Life Inventory (PedsQL) in the Netherlands is limited and Dutch reference data are lacking. Aim of the current study is to collect Dutch reference data of the PedsQL and subsequently assess reliability, socio-demographic within-group differences and construct validity.

**Methods:**

In this study the PedsQL was administered to Dutch children aged 5 to 18 years. A socio-demographic questionnaire was completed as well. The sample consisted of three age groups: 5-7 years (parent proxy report), 8-12 years and 13-18 years (child self report). Analysis was performed with SPSS 16.0.2. A reliability analysis was done using Cronbach's alpha coefficient. Socio-demographic within-group differences were assessed by means of an ANOVA with post hoc Bonferroni correction and t-tests. Subsequently, construct validity was determined by t-tests and effect sizes.

**Results:**

For 496 children PedsQL reference data were collected. PedsQL total scores were 84.18 (group 5-7), 82.11 (group 8-12) and 82.24 (group 13-18). Internal consistency coefficients ranged from .53 to .85. Socio-demographic within-group differences demonstrated that, in group 8-12, children of parents born in the Netherlands had significantly lower scores on several PedsQL subscales, compared to children of parents born in another country. With respect to construct validity, healthy children of group 5-7 and 13-18 scored significantly higher than children with a chronic health condition on all subscales, except for emotional functioning. In group 5-7, the PedsQL total score for healthy children was 85.31, whereas the same age group with a chronic health condition scored 78.80. Effect sizes in this group varied from 0.58 to 0.88. With respect to group 13-18, healthy children obtained a PedsQL total score of 83.14 and children suffering from a chronic health condition 77.09. Effect sizes in this group varied from 0.45 to 0.67. No significant differences were found in group 8-12 regarding health.

**Conclusion:**

The Dutch version of the PedsQL has adequate psychometric properties and can be used as a health related quality of life instrument in paediatric research in the Netherlands.

## Background

The World Health Organization defines quality of life (QOL) as "individuals' perceptions of their position in life, in the context of the culture and value systems in which they live, and in relation to their goals, expectations, standards and concerns" [1]. The concept of health related quality of life (HRQOL) refers to the impact of health and illness on an individual's QOL [2]. It is generally accepted that HRQOL is a multidimensional construct incorporating at least three broad domains: physical, psychological and social functioning [3].

To assess HRQOL in children, different questionnaires are needed for different age groups. Comparison of HRQOL outcomes in children from different age groups or over time is complicated. It is important to consider the use of proxy ratings as substitutes for ratings made by children themselves. A case in point would be when a child is too young or too ill to complete the questionnaires himself [2].

In recent years, several HRQOL questionnaires for children were developed. Examples of questionnaires which can be administered to both healthy and chronically ill children are the TAPQOL [4], TACQOL [5], Kidscreen [6] and CHQ [7]. These are referred to as generic questionnaires. For children with a chronic health condition, disease specific questionnaires were designed as well, e.g. the Disabkids [8].

The Pediatric Quality of Life Inventory 4.0 Generic Core Scale (PedsQL) measures HRQOL in both healthy and chronically ill children. Besides a 23-item generic module, the PedsQL has several disease specific modules, for instance for asthma [9], cancer [10] and cerebral palsy [11]. The PedsQL encompasses four domains of HRQOL: physical, emotional, social and school functioning and covers a broad age range, including child self-reports as well as parent proxy-reports. Both comprise age versions 5-7, 8-12 and 13-18 years; parent proxy-report also incorporates ages 2-4 years. Feasibility, reliability and validity of the PedsQL are found to be good [12]. Although translated and studied before [13], knowledge about psychometric properties of the PedsQL in the Netherlands is limited and Dutch reference data are lacking.

Aim of the current study is to collect Dutch reference data of the PedsQL and subsequently assess reliability, socio-demographic within-group differences and construct validity of this instrument in the Netherlands.

## Methods

### Procedure

The PedsQL Generic Core Scale was administered to Dutch children aged 5 to 18 years between October 2006 and January 2007. Stratification criteria for school were geographic location (urban, suburban and rural), percentage of migrant children (low, middle and high) and level of education (low, middle and high).

Parents received a letter concerning the purpose and procedure of the study. Confidential processing of personal information and collected data was explained and guaranteed. Furthermore, a socio-demographic questionnaire and a form for non-participants were enclosed. Parents were requested to complete the socio-demographic questionnaire and send it back to school. If parents did not want to participate they were asked to fill out the non-participants form.

Three age versions of the PedsQL were administered in this study: parent proxy-report ages 5-7 years (group 5-7), child self-report ages 8-12 years (group 8-12) and ages 13-18 years (group 13-18). For the purpose of this study, the PedsQL was converted into two computer programs: one stand alone computer version (group 5-7 and 8-12) and an internet version (group 13-18). Layout of both programs was identical and resembled the paper version as much as possible, except that questions were presented one at a time instead of all at once and missing values were not accepted.

Parents and children from group 5-7 and group 8-12 were invited to school to complete the PedsQL on a laptop. Group 13-18 filled out the PedsQL via internet in a computer room at school. It concerned a simultaneous procedure, in which several children participated at the same time. Each child logged in on a website with a unique personal number. In all groups, two psychology students clarified the procedure and made sure children did not interfere with classmates. During all administrations PedsQL Manual instructions were observed.

The study was approved by the Medical Ethics Committee of the Amsterdam Medical Centre, the Netherlands.

### Measures

#### PedsQL

The PedsQL accentuates perceptions of children and reflects their universal concerns on four subscales: physical (8 items), emotional (5 items), social (5 items) and school functioning (5 items). Each of the 23 items states a problem, for example "difficulty walking". The parent or child indicates on a 5-point Likert scale to what extent the child had difficulties with that problem. Answering options are never (0), almost never (1), sometimes (2), often (3) and almost always (4). Each answer is reversed scored and rescaled to 0-100 scale (0 = 100, 1 = 75, 2 = 50, 3 = 25 and 4 = 0). A score of 100 represents the best quality of life possible, a score of 0 the worst. Completion time is about 5-10 minutes. Parent proxy-reports are a parallel version of the child self-reports. Differences are the use of age appropriate language and first- or third-person tense. The PedsQL refers to two different periods of time: the past month or the past week [12]. The past week version was used in this study.

### Socio-demographic questionnaire

The socio-demographic questionnaire consists of five items concerning the parent (date of birth, gender, country of birth, education and employment) and six items with respect to the child (date of birth, gender, presence of a chronic disease, contact with general practitioner and/or medical specialist over the past year, received psychological care over the past year).

### Non-participants form

The non-participants form is a brief list on which parents and children can indicate they are not interested in participating in this study. Parents are additionally requested to complete the five previously mentioned socio-demographic questionnaire items.

### Statistical analysis

Data were analyzed with SPSS 16.0.2. Differences between participants and non-participants with respect to gender, country of birth and employment were analysed by means of Chi^2 ^test. Age differences were examined with a t-test; a Mann-Whitney test was performed for education. Difference was made between low education (no education, primary school and primary vocational education) middle education (secondary school and secondary vocational education) and high education (higher vocational education and university). PedsQL scores were computed according to the PedsQL Manual and a reliability analysis was done using Cronbach's alpha coefficient based on average inter item correlation (according to SPSS 16.0.2). Socio-demographic within-group differences regarding PedsQL scores were assessed by means of an ANOVA with post hoc Bonferroni correction (p < .0167) for age group and education and t-tests for gender, country of birth and employment. Subsequently, construct validity was determined by t-tests and effect sizes (es), analysing differences in PedsQL scores (per age group) between healthy and chronically ill children and between children that did or did not visit their general practitioner, medical specialist or psychologist over the past year. Effect sizes were calculated dividing the difference in mean scores between the chronically ill and healthy group by the standard deviation of the healthy group. For the group of children that visited their general practitioner, medical specialist or psychologist effect sizes were determined thru the same formula, with the chronically ill group being replaced by respectively the group of children that visited the general practitioner, the medical specialist or psychologist. Effect sizes up to 0.2 were considered to be small, effect sizes of about 0.5 moderate and effect sizes of about 0.8 large [14].

## Results

### Participants

In total, 891 children from Amsterdam and surrounding regions were approached. They attended four elementary schools (three suburban, one urban), four high schools (one rural, one suburban, two urban) and one school for vocational education (urban). 544 (61.1%) parents gave informed consent, 53 (5.9%) declined participation and 294 (33.0%) parents did not respond. Eventually, the PedsQL was completed by 496 (55.7%) participants.

Parent gender, ethnicity and education were not evenly distributed among participants and non-participants. There were significantly less fathers in the participating group than in the non-participating group (p < .01). In addition, more participating parents were born in the Netherlands (p < .01) and a larger percentage of the participating parents was highly educated (p < .05), compared to non-participants. Socio-demographic data are summarized in Table 1 and 2.

**Table 1:** Socio-demographic data of participants and non-participants

	**Participants**			**Non-participants**^1^			
			
***Child***	**N**	**Mean**	**SD**	**N**	**Mean**	**SD**	**p value**
Age	496	11.80	3.20	81	11.44	2.75	.33
	N	%		N	%		
		
Gender (boys)	227	45.8		35	43.8		.74
***Parent***^2^	N	Mean	SD	N	Mean	SD	

Age	479	44.35	5.73	82	43.16	6.29	.09
	N	%		N	%		
		
Gender (male)	92	19.4		31	36.5		<.01**
Ethnicity (Dutch)	414	85.9		53	62.4		<.01**
Education (high)	230	48.4		30	38.0		<.05*
Employment	349	72.9		52	62.7		.06

^1^Children and parents that refused participation and completed the non-participants form; or children and parents that consented to participation and filled out the socio-demographic questionnaire, but eventually did not complete the PedsQL due to circumstances (e.g. illness, dental visit).^2^Socio-demographic data of parent that completed the questionnaire. * Sign: p < .05, ** Sign: p < .01.

**Table 2:** Socio-demographic data of participants per age group

	**Age group** **5-7**		**Age group** **8-12**		**Age group** **13-18**	
			
***Child***	**M**	**SD**	**M**	**SD**	**M**	**SD**
Age	6.67	0.73	11.27	1.35	14.98	1.20
	N	%	N	%	N	%
	
Total	92	18.5	219	44.2	185	37.3
Gender (boys)	43	46.7	104	47.5	80	43.2
Chronic health condition^1^	14	15.6	26	11.9	25	14.5
Contact general practitioner	54	60.7	91	41.9	88	50.9
Contact medical specialist	20	22.2	55	25.2	44	25.6
Psychological care	1	1.1	23	10.7	14	8.2
***Parent***^2^	M	SD	M	SD	M	SD

Age	40.58	4.77	43.67	5.66	47.23	4.78
	N	%	N	%	N	%
	
Gender (male)	15	16.7	37	17.4	40	23.3
Ethnicity (Dutch)	87	95.6	177	81.2	150	86.7
Education (high)	57	64.0	97	44.7	76	45.0
Employment	61	67.8	158	72.5	130	76.0

^1^Most common conditions in total Dutch sample were: asthma (36.4%), congenital defect (13.6%), skin disease (6.1%) and migraine (6.1%). ^2^Socio-demographic data of parent that completed the questionnaire.

### PedsQL scores and reliability

PedsQL scale scores ranged from 74.59 (group 13-18, school functioning subscale) to 89.38 (group 13-18, social functioning subscale). Total PedsQL score for group 5-7 was 84.18, for group 8-12 82.11 and group 13-18 scored 82.24 (Table 3).

**Table 3:** PedsQL scale scores

			**PedsQL Scale Scores**	
**Age group**	**N**	**PedsQL subscale**	**M**	**SD**

5-7	92	Total score	84.18	8.96
		Physical health	87.33	10.25
		Psychosocial health	82.50	9.89
		Emotional functioning	75.82	13.33
		Social functioning	86.20	12.21
		School functioning	85.49	11.60
				
8-12	219	Total score	82.11	8.87
		Physical health	84.87	9.30
		Psychosocial health	80.63	10.31
		Emotional functioning	77.05	13.66
		Social functioning	86.14	12.30
		School functioning	78.70	12.00
				
13-18	185	Total score	82.24	9.15
		Physical health	86.01	9.77
		Psychosocial health	80.23	10.18
		Emotional functioning	76.70	15.20
		Social functioning	89.38	11.56
		School functioning	74.59	13.16

Internal consistency coefficients ranged from .53 to .85 (Table 4). Most subscales had an alpha higher than .60. Overall, reliability was comparable across age groups, with the total score being the most reliable and the school functioning subscale the least.

**Table 4:** PedsQL scales: internal consistency reliability

**PedsQL subscale**	**Age group 5-7** **α**	**Age group 8-12** **α**	**Age group 13-18** **α**
Total score	.85	.82	.85
Physical health	.69	.61	.69
Psychosocial health	.82	.79	.80
Emotional functioning	.70	.61	.74
Social functioning	.73	.67	.76
School functioning	.59	.53	.62

### Socio-demographic within-group differences

#### Age

Results indicate a significant difference between age groups for the subscale school functioning (p < .01). Post hoc tests demonstrated that group 5-7 scored higher than group 8-12 (mean difference = 6.79, p < .001) and group 13-18 (mean difference = 10.89, p < .001). In addition, group 8-12 had higher scores than group 13-18 (mean difference = 4.10, p < .01).

#### Gender

There was a significant gender effect on the emotional subscale in age group 8-12; here, boys scored higher than girls (mean difference = 4.24, p < .05).

#### Ethnicity

In group 8-12, children of parents born in the Netherlands had a significantly lower total (mean difference = 3.94, p < .05), psychosocial (mean difference = 4.63, p < .01), emotional (mean difference = 5.39, p < .05) and school functioning score (mean difference = 4.31, p < .05) compared to children of parents born in another country.

#### Education

Differences were found with respect to education in group 13-18. Post hoc tests revealed that children of low educated parents scored higher than children of parents with a middle education on the total (mean difference = 6.21, p < .0167), psychosocial (mean difference = 7.69, p < .01), social (mean difference = 8.46, p < .01) and school subscale (mean difference = 8.99, p < .0167) of the PedsQL. Within the social domain scores of children from parents with a high education were significantly higher compared to children of parents that had a middle education (mean difference = 5.43, p < .0167).

#### Employment

For none of the age groups and subscales differences were found regarding employment.

### Construct validity

Table 5 contains PedsQL scores of children with and without a chronic health condition. Healthy children in groups 5-7 and 13-18 scored significantly higher on all subscales, except for emotional functioning, than peers with a chronic health condition. Effect sizes varied from moderate to high. No significant differences were found in group 8-12.

**Table 5:** PedsQL scales: construct validity

		**Healthy sample**			**Chronic health condition**			
				
**Subscale**	**N**	**Mean**	**SD**	**N**	**Mean**	**SD**	**Effect size**	**p value**
Age group 5-7	76			14				
Total score		85.31	8.59		78.80	9.58	0.76	<.05*
Physical health		88.65	9.13		80.58	13.71	0.88	<.01**
Psychosocial health		83.53	9.70		77.86	10.22	0.58	<.05*
Emotional functioning		76.38	13.80		73.93	11.30	0.18	.53
Social functioning		87.76	11.70		80.00	12.56	0.66	<.05*
School functioning		86.45	10.76		79.64	15.00	0.63	<.05*
								
Age group 8-12	192			26				
Total score		82.31	8.83		80.64	9.32	0.19	.37
Physical health		85.25	8.85		82.21	12.14	0.34	.12
Psychosocial health		80.75	10.34		79.81	10.43	0.09	.67
Emotional functioning		76.85	13.76		78.85	13.21	-0.15	.49
Social functioning		86.51	12.24		83.27	12.80	0.26	.21
School functioning		78.88	11.90		77.31	13.13	0.13	.53
								
Age group 13-18	148			25				
Total score		83.14	8.99		77.09	9.40	0.67	<.01**
Physical health		86.76	9.21		81.00	12.00	0.63	<.01**
Psychosocial health		81.21	10.22		75.00	9.56	0.61	<.01**
Emotional functioning		77.53	15.01		71.40	16.62	0.41	.07
Social functioning		90.14	11.37		83.40	12.97	0.59	<.01**
School functioning		75.95	12.68		70.20	15.17	0.45	<.05*

* Significant p < .05, ** Significant p < .01.

Children also scored significantly lower on several PedsQL subscales when they had contact with their general practitioner over the past year. In group 5-7, this was the case for social functioning (p < .05, es = 0.49) and in group 8-12 for the total (p < .05, es = 0.31), physical (p < .05, es = 0.32) and school functioning subscale (p < .05, es = 0.37). Children belonging to group 13-18 obtained lower scores on all subscales except for school functioning: total (p < .01, es = 0.51), physical (p < .01, es = 0.70), psychosocial (p < .05, es = 0.37), emotional (p < .05, es = 0.34) and social functioning (p < .05, es = 0.36).

In some cases, children also had lower PedsQL scores when they had contact with a medical specialist over the past year. For groups 5-7 and 8-12 this difference was observed on the school functioning subscale (both p < .05, respectively es = 0.65 and es = 0.31). In group 13-18 the difference was apparent on more subscales: total (p < .05, es = 0.46), physical (p < .05, es = 0.42), psychosocial (p < .05, es = 0.42) and school (p < .05, es = 0.39).

Finally, in some cases psychological care was an indicator for lower PedsQL scores as well. In group 8-12, a difference was measured for the total (p < .05, es = 0.51), physical (p < .05, es = 0.46) and psychosocial subscale (p < .05, es = 0.46). In the oldest group 13-18, these children obtained lower scores with respect to total (p < .05, es = 0.67), psychosocial (p < .05, es = 0.68) and social functioning (p < .01, es = 0.80).

## Discussion

In this study, reference data of the Dutch PedsQL Generic Core Scale were collected and psychometric properties were assessed in a sample of 496 children aged 5 to 18 years and their parents. The psychometric properties of the PedsQL in the Netherlands range from sufficient to good.

The PedsQL reference scores obtained in this study are an adequate representation of the general Dutch population. According to Statistics Netherlands [15] in 2008 80.4% (of the total population) were from Dutch origin and 77.9% (of population aged 45-55 years) were employed. These figures are comparable to the socio-demographic data from our study. Furthermore, it is not uncommon [16] to find a large percentage of highly educated parents (48.4%) in the current study, compared to the Dutch working population (33.5%). It is likely that highly educated parents are better aware of the necessity of this type of research, and thus be more willing to participate. During data collection we experienced that willingness to take part in the study seemed lower on schools with lower educational levels and higher percentages of migrant children. Possibly, parents not born in the Netherlands experienced language problems and for this reason did not fully understand the information letter to participate in the study. These findings are supported by the fact that there were fewer parents with a high education and Dutch ethnicity in the non-participants group, compared to the group that did participate.

The Dutch sample shows the same trend in reliability across subscales as the US [12], however with slightly lower alphas. In both samples, the total score appears to be most reliable and subscale school functioning least. A possible clarification for the lower Dutch reliability figures could be the difference in sample size. The US sample ranges from 1159 to 2674 children per age group; the Dutch from 92 to 219. More over, the US sample included children from the State Children's Health Insurance Program (SCHIP) that provides health insurance coverage to uninsured children from low-income families [17]. In the current study a stratified sampling technique was applied in order to create a sample as diverse as possible, which can be generalized to the overall Dutch population. Bastiaansen et al [13] have investigated the psychometric properties of the PedsQL in the Netherlands as well. In their study a sample (n = 74) from the general Dutch population (6-18 years) was compared with 310 children referred for psychiatric problems: alpha's were in concordance with Varni et al [12], however, their sample size was very small.

Regarding the socio-demographic within-group differences, this study demonstrates a gender difference in group 8-12 on the emotional subscale with girls obtaining lower scores than boys. Similar results in adolescent girls have been reported by Reinfjell et al [18]. 

Salient finding is that country of birth of the parent had an effect on PedsQL scores in group 8-12. Children of parents born in the Netherlands scored significantly lower on all subscales except physical functioning. Previous studies only correspond partly with this result. For instance in the US, White together with Asian children scored higher than Hispanic and Black children [12]. Important to note is that we only collected data regarding country of birth of the parent, and not country of birth of the child. Moreover, this difference was not found in groups 5-7 and 13-18.

Results of our study indicate a relationship between educational level and PedsQL scores in group 13-18: children of parents with a low education perceived a significantly better HRQOL. This phenomenon is difficult to explain, since previous research mainly pointed out that high quality of life scores were related to high parental education, or that education had no effect at all [19,20].

Findings on parental employment are also notable: having a job had no influence on the child's HRQOL in our sample. Previous research has shown that children with low socioeconomic status (SES) functioned worse than children from middle SES backgrounds [21]. Employment and SES are not exchangeable, though. Having a job does not necessarily implicate a middle or high SES.

With respect to construct validity, this study demonstrates that the Dutch PedsQL version differentiates between children with and without a chronic condition in group 5-7 and 13-18. Differences in group 8-12 were not significant - however, except for the emotional functioning subscale, healthy children obtained higher scores than their chronically ill peers. Although several studies have shown that the PedsQL differentiates between children with and without a chronic health condition [9,12,13] it is not exceptional to find adequate functioning for chronically ill children [22]. Another explanation could be that severely ill children did not take part in our study, because they were not present at the time of administration due to illness or that parents did not want to burden them with participation. A further possible reason might be the fact that the presence of a chronic health condition in our sample was determined by the parent and not diagnosed by a physician. Physician-diagnosed chronic health conditions are often stricter than those reported by the parent. Therefore, the 8-12 year old chronic health condition sample in our study might not be completely representative of children with more serious chronic diseases.

Limitations of the study need to be taken into account. First, a considerable number of children were approached, which eventually resulted in nearly 500 participants. However, considering the different age groups and the socio-demographic within-group differences, sample sizes per group were relatively small. Furthermore, it is possible that presenting PedsQL items one at a time on a computer - with missing values not being allowed - could have some psychometric implications. Digital administration of the PedsQL has demonstrated equivalent measurement properties to the paper version [23], yet in the study of Varni et al (2008) each PedsQL scale was depicted on a separate screen and participants had the option to skip items. The fact that this possibility was lacking in the current study could have forced participants to choose an answer that did not really apply to them. Nonetheless, this probably concerns a minimum of items since Varni et al (2008) also demonstrated similar (low) percentages of missing-item responses of digital and paper version PedsQL. Additionally, it would have been interesting to have examined all age versions of the PedsQL involving all regions of the Netherlands, but this was unfeasible for the purpose of our study. Therefore, we recommend that future research with respect to the PedsQL in the Netherlands should include more regions of the country and incorporate the remaining PedsQL versions.

## Conclusion

We conclude that the Dutch version of the PedsQL has adequate psychometric properties and can be used as an HRQOL instrument in pediatric research in the Netherlands.

## Competing interests

The authors declare that they have no competing interests.

## Authors' contributions

VE contributed to the concept and design of the study, analysed the data and drafted the manuscript. MMH carried out the data acquisition and helped analysing. SBD, HMK and MAG contributed to the concept and design of the study and revised the manuscript. All authors read and approved the manuscript.

## Pre-publication history

The pre-publication history for this paper can be accessed here:


